# Academic and non-academic life stressors and their impact on psychological wellbeing of medical students

**DOI:** 10.3934/publichealth.2021046

**Published:** 2021-08-10

**Authors:** Ahmad A Mirza, Hammam Baarimah, Mukhtiar Baig, Abdulrahim A Mirza, Mohammed A Halawani, Ghada M Beyari, Khalid S AlRaddadi, Mahmoud Alreefi

**Affiliations:** 1 Department of Otolaryngology, Head and Neck Surgery, Faculty of Medicine in Rabigh, King Abdulaziz University, Jeddah, Saudi Arabia; 2 Psychiatrist, Makkah Healthcare Cluster, Ministry of Health, Saudi Arabia; 3 Department of Clinical Biochemistry/Medical Education, Faculty of Medicine, Rabigh, King Abdulaziz University, Jeddah, Saudi Arabia; 4 Department of Surgery, Division of Urology, Ministry of National Guard - Health Affairs, Jeddah, Saudi Arabia; 5 Faculty of Medicine, Umm Al-Qura University, Makkah, Saudi Arabia; 6 Faculty of Medicine, Umm Al-Qura University, Makkah, Saudi Arabia; 7 Department of Primary Health Care, National Guard Health Affairs, King Saud bin AbdulAziz University for Health Sciences, Jeddah, Saudi Arabia; 8 Department of Otolaryngology, Head and Neck Surgery, Faculty of Medicine in Rabigh, King Abdulaziz University, Jeddah, Saudi Arabia

**Keywords:** anxiety, depression, psychological stressors, public health, students

## Abstract

**Objectives:**

Among medical students, depression, anxiety, and stress (DAS) are key public wellbeing challenges that require epidemiological research. We aimed to evaluate potential sources of these psychological disturbances and assess the contribution of academic and non-academic life stressors in psychological morbidity among medical students.

**Methods:**

This exploratory questionnaire-based survey was conducted in a Saudi Arabian public sector medical college. A total of 231 medical students were enrolled and completed the depression, anxiety, and stress scale-21 (DASS-21) questionnaire.

**Results:**

More than half of the medical students, 129 (55.8%), had depression, 106 (45.9%) students had anxiety, and 87 (37.7%) students had stress. Academic achievement was the largest explanatory factor for depression and stress, whereas bodily appearance constituted the largest explanatory factor of anxiety among the study sample. Academic and non-academic stressors score was significantly associated with depression (adjusted Odds Ratio, aOR = 1.13, 95% CI 1.07–1.19), anxiety (aOR = 1.07, 95% CI 1.03–1.12), and stress (aOR = 1.12, 95% CI 1.08–1.17).

**Conclusions:**

Medical students have a high incidence of negative emotional states. These negative psychological states were explained by academic achievement and bodily appearance. The studied stressors influenced medical students' psychological wellbeing.

## 1. Introduction

The symptoms of depression, anxiety, and stress (DAS) among the student population are one of the causes of public concern in Western societies [1]. Several studies worldwide have found that symptoms of psychiatric disorders are common among medical students than students of other faculties and people in the general population [2]–[4]. Medical students may suffer from psychological distress that impairs their academic progress and success and contributes to medical school withdrawal. It may also increase the risk of substance abuse as a coping method to deal with the increased stress [2].

A longitudinal epidemiological report showed a high incidence of major depressive disorder and increased prevalence of the disorder over the years [5]. A systematic analysis reported approximately one-third of the university students across the globe had symptoms of depressive disorders, and those were substantially higher than the recorded rates among general populations [6]. Medical students tend to experience a high level of symptoms of depression. Its incidence increases by 17% from the first year to the third year, indicating that depression is accumulative, and the rate of the disease could rise higher if not well managed [7]. Depression wss found to be as high as 83% among Saudi medical students [8]–[10]. A study has shown depression in 45% of students and reported several predictors of depression, such as the presence of psychological ailment, recent death of a family member, social lifestyle, and contentment with teaching staff were predictors of depression [11]. Another study demonstrated high prevalence of depressive symptoms (39.27%) among the medical students [12]. A systematic review from Saudi Arabia reported several academic and non-academic factors related to psychological distress such as workload pressure, attendance, multiple assessments, lengthy curriculum, academic achievements, trust concerns, monetary problems, irregular eating patterns, and fear of the future [13]. A recent systematic review reported 30.9% to 77.6% prevalence of depressive symptoms among Saudi Arabian medical students [14].

Several types of research across the globe documented the incidence of anxiety symptoms among medical students. A systematic analysis targeted medical students in the United Kingdom, Europe, and elsewhere in the English-speaking world outside North America showed that anxiety prevalence ranged from 7.7% to 65.5% [15]. Anxiety was estimated to be present in 34.9% of Saudi medical undergraduates [16]. In addition, anxiety was shown to be more prevalent in 1^st^ year female medical students (89.7%) than in male students (60%) [17]. A systematic review study reported 34.9% to 65% prevalence of anxiety among students. Female medical students were more anxious than male medical students. Depression and anxiety symptom ratings have a positive association [18]. A study from Makkah reported higher levels of depression and anxiety symptoms, and loss of control among male medical students compared to females. This study also reported that students with lower levels of depressive symptoms had higher GPAs. These findings emphasize the importance of dealing with mental health disorders and removing risk factors [19].

Stress symptoms are common psychological problems experienced by medical students throughout their undergraduate courses [10],[20]–[22]. It causes poor academic performance and can be linked to emotional and physical illnesses [23]–[25]. Stress prevalence ranges from 28.9% to 70.9% among Saudi medical students [10],[26],[27]. Stress and depression were found in 80.3% and 38.7% of the students, respectively [28]. A Saudi study from Jazan University reported a high rate of symptoms of depression (53.6%), anxiety (65.7%), and stress (34.3%) [29]. Another Saudi study observed a high prevalence of depression (67.4%), anxiety (79.7%), stress (64%), and low self-esteem (23.4%). A significant inverse relationship between self-esteem and symptoms of depression, anxiety, and stress was also reported [30].

Many factors may increase the vulnerability of students to depression, anxiety, and stress. Several causes of depression have been mentioned, including changes in lifestyle, financial stressors, family relationship changes, academic worries with post-graduation life [6]. A study highlighted some factors concerned with college students, including academic performance, load to succeed, and post-graduation plans, besides other socio-demographic factors such as living in students' residence facilities [31]. Medical students and their wellbeing are influenced by many stressors, including growing learning pressures, frequent tests, and curriculum burden [32]. Additionally, curricular factors, personal life events and the learning environment can predispose these vulnerable students to psychological illnesses [33]–[35].

The mental health of medical students represents important public health challenges that require epidemiological research. In addition to their academic and social pressure, students have to cope with psychosocial changes related to their personal lives and plan for their future careers. Therefore, we aimed to assess levels of perceived psychological distress related to various academic and non-academic parameters. Furthermore, the extent of involvement of those academic and non-academic parameters in psychological morbidity among medical students was also investigated.

## 2. Materials and methods

This exploratory questionnaire-based survey was carried out at Umm Al-Qura University (UQU), Makkah, Saudi Arabia, after receiving approval from the Institutional Ethical Committee at UQU Faculty of Medicine. We included male and female students from the academic year of 2019. A total of 1317 students (male = 630; female = 658; male to female ratio = 1:1.04) were registered in the second to sixth year at the Faculty of Medicine during the study period. The first-year medical students were excluded from the current study as they are not administered by the Faculty of Medicine. A self-administered anonymous questionnaire was distributed with five main components:

1) Socio-demographics (e.g., age, gender, marital status, hometown, family income)

2) Academic profile (e.g., academic year, GPA, number of registered university hours)

3) Lifestyle factors (e.g., smoking, energy drinks, physical exercise, eating habits)

4) A panel of academic and non-academic potential stressors

5) Depression, anxiety and stress scale—21 items (DASS-21)

The DASS-21 has been proved to be a valid and reliable assessment of the dimensions of depression, anxiety, and stress independently, as well as a more general measure of psychological distress [36]. We used the validated DASS-21 screening instrument to assess the prevalence of DAS symptoms among the study participants. Furthermore, the DASS-21 is a screening tool rather than a diagnostic instrument [37]. Decisions based on specific score profiles, on the other hand, should be made only by competent clinicians after a thorough clinical assessment [37]. The DASS-21 was chosen because it is an uncomplicated, dependable, and validated tool that can be used for effective screening and assessment. Several investigations in the literature have validated this scale and found that every subscale has a good internal consistency (Cronbach's alpha) [36],[38],[39]. Sinclair et al. (2011) reported satisfactory Cronbach's alpha for subscales (DASS21-D subscale 0.91; DASS21-A subscale 0.80; and DASS21-S subscale 0.84) [38]. Daza et al. also demonstrated good Cronbach's alpha values for subscales (DASS21-D subscale 0.93; DASS21-A subscale 0.86; and DASS21-S subscale 0.91) [39]. Similarly, Crawford & Henry (2003) reported good internal consistency values (DASS21-D subscale 0.88; DASS21-A subscale 0.82; and DASS21-S subscale 0.90) [36].

DASS-21 is a self-reported scale that consists of 21 statements, 7 items in 3 subscales (DAS subscale). Students had to mark their feeling based on the last week on a 4-point (0–3) scale, (0: Did not apply to me at all, 1: Applied to me to some degree or some of the time, 2: Applied to me to a considerable degree or a good part of the time, 3: Applied to me very much or most of the time). The total score of each subscale was calculated by summing the scores of the relative items. Due to the use of the short version of DASS-42, the total score of each subscale was multiplied by 2. According to the DASS manual, the three emotional states can be categorized into “mild,” “moderate,” “severe,” and “extremely severe” based on cut-off values [37].

A panel of academic and non-academic potential stressors were evaluated against DAS. These stressors were studied and reported in the literature: “1) university environment, 2) science content, 3) efforts to studying, 4) relationship with college mates, 5) relationship with teachers, 6) academic achievement, 7) health status, 8) economic status, 9) home environment, 10) relationship with family, 11) emotional life, 12) relationship with friends, 13) bodily appearance” [31],[40]. Study participants were asked to rate the magnitude of each of those elements as a source of psychological distress on a scale of 1 (not at all) to 5 (extreme amount). A summary score indicating the level of perceived psychosocial distress related to those academic and non-academic stressors was computed for each student by taking the average of those stressors scores. The reliability coefficient of this part of the questionnaire was calculated (Cronbach's alpha = 0.89), indicating a high internal consistency. Face validity of this part of the questionnaire was confirmed by a research consultant and biostatistician.

Data was analyzed on Stata v13 software (Stata Corp. 2013. Stata Statistical Software: Release 13. College Station, TX: Stata Corp LP). Descriptive statistics were computed, and frequencies, along with percentages, were used to represent the categorical variables. Histogram and the Shapiro-Wilk analysis were used to test the normality. Mean and standard deviations were computed to express normally distributed numerical variables. Non-parametric data were presented as medians and interquartile ranges (IQRs) at the 25^th^ and 75^th^ percentiles. Stepwise multiple linear regression analyses were carried out where depression, anxiety, and stress (as continuous outcomes) were the dependent variables, and the academic and non-academic stressors (questions 1–13) were the independent variables. Subsequent multivariate binary logistic regression analyses were conducted to avoid confounding effects of socio-demographics, related academic factors and lifestyle, and to predict the impact of academic and non-academic stressors on DAS (the dependent variables). The dependent variables were dichotomous variables with “normal” vs. “abnormal” categories. The academic and non-academic stressors score was kept in the model regardless of its significance level. All other statistically significant variables at p < 0.1 in the univariate analysis were included in the multivariate logistic regression. The multivariate logistic analysis was conducted using the backward stepwise method, with an entry p-value of 0.10 and a removal p-value of 0.101 for variable selection. The p-value was considered to be significant at <0.05.

## 3. Results

**Figure 1.**
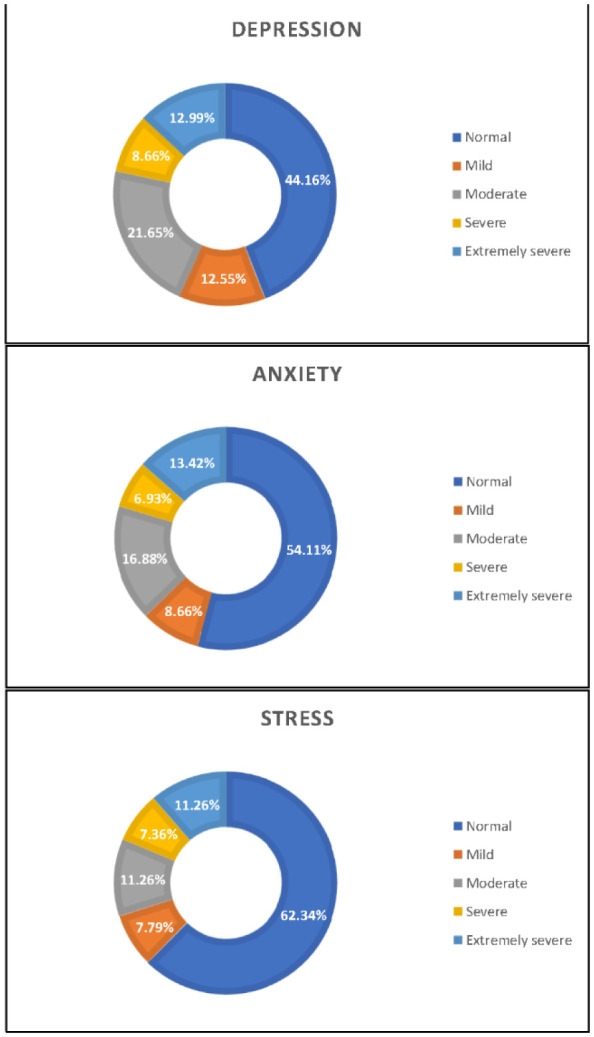
Prevalence and severity of DAS among medical students.

**Figure 2.**
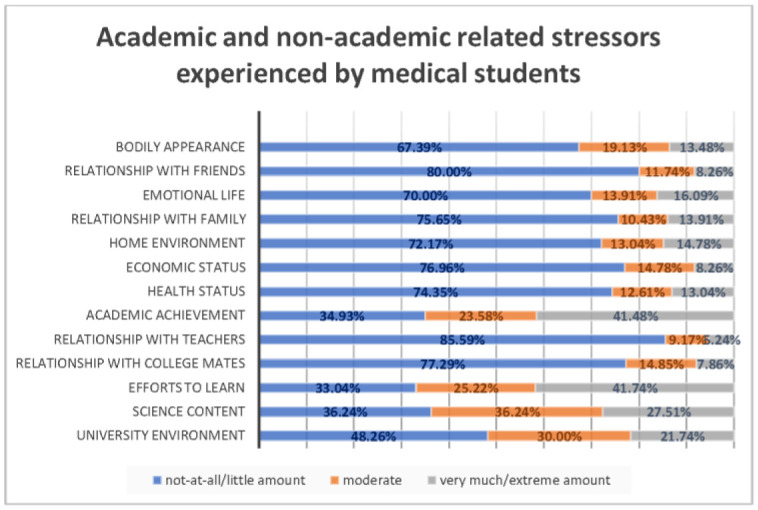
Levels of perceived psychological distress with regards to academic and non-academic stressors.

The mean age of the study participants was 21.67 ± 1.56. There were 106 (45.89%) males and 125 (54.11%) females, and most participants were unmarried 224 (96.97%) and lived in urban area 224 (96.97%). Socio-demographic characteristics, academic profile, and lifestyle characteristics of the study participants are presented in Table 1. A total of 129 (55.84%) medical students had depression, 106 (45.89%) students were found to have anxiety, and 87 (37.66%) students had stress (Figure 1). Table 2 and Figure 2 illustrate the summary results of each academic and non-academic stressors. A large number of students perceived academic achievement (41.48%) and efforts to learn (41.74%) as causes of excessive psychological distress.

**Table 1.** Socio-demographic, academic and lifestyle characteristics of the study participants.

	Variables		N (%)
Socio-demographic Characteristics	Age	mean (SD)	21.67 (1.56)
	Gender	Male	106 (45.89)
		Female	125 (54.11)
	Marital status	Not married	224 (96.97)
		Married	7 (3.03)
	Do you have children?	Yes	0 (0)
		No	231 (100)
	Hometown	Urban	224 (96.97)
		Rural/Countryside	7 (3.03)
	Father's educational level*	Illiterate	2 (0.87)
		Primary-intermediate	28 (12.17)
		Secondary	62 (26.96)
		University+	138 (60.00)
	Mother's educational level	Illiterate	9 (3.90)
		Primary-intermediate	29 (12.55)
		Secondary	45 (19.48)
		University+	148 (64.07)
Socio-demographic Characteristics	Residence place during study period	Students' residence facility	9 (3.90)
		Living with family	222 (96.10)
	Time from home to university	<15 minutes	57 (24.68)
		15–30 minutes	127 (54.98)
		30–60 minutes	42 (18.18)
		>60 minutes	5 (2.16)
	Family monthly income (in Saudi Riyals)*	<5000	6 (3.55)
		5000–10000	32 (18.93)
		>10000–20000	74 (43.79)
		>20000	57 (33.73)
	Working during the study period*	Yes	16 (6.96)
		No	214 (93.04)
	Parent's status	Separated or dead	45 (19.48)
		Living together and married	186 (80.52)
	Family conflicts at home	Composed/stable	159 (68.83)
		Minor conflicts	61 (26.41)
		Major conflicts	11 (4.76)
	Family responsibilities	Simple	137 (59.31)
		Moderate	80 (34.63)
		Burdensome	14 (6.06)
Academic Profile	Number of weekly registered hours*	median (IQR)	20 (20-30)
	Academic level	Pre-clinical (1^st^, 2^nd^ or 3^rd^ yr)	89 (38.53)
		Clinical (4^th^, 5^th^ or 6^th^ yr)	142 (61.47)
	Did you match with the first choice in admission? *	Yes	197 (86.40)
		No	31 (13.60)
	Failed in module(s)*	Yes	12 (5.24)
		No	217 (94.76)
	GPA*	median (IQR)	3.42 (3.09–3.79)
	Number of daily hours of learning at home	<1 hour	21 (9.09)
		1–2 hours	86 (37.23)
		>2–4 hours	90 (38.96)
		>4 hours	34 (14.72)
Lifestyle Characteristics	Have you been diagnosed with a psychiatric condition?	Yes	15 (6.49)
		No	216 (93.51)
	Following-up with a psychiatrist or psychologist	Yes	10 (4.33)
		No	221 (95.67)
	Have you been diagnosed with other chronic medical illness?	Yes	27 (11.69)
		No	204 (88.31)
	Do you take medication(s) regularly?	Yes	20 (8.66)
		No	211 (91.34)
	Do you smoke currently? *	Yes	23 (10)
		No	207 (90)
	Coffee or tea	Never	12 (5.19)
		Occasionally	60 (25.97)
		Once daily	83 (35.93)
		≥2 times daily	76 (32.90)
Lifestyle Characteristics	Energy beverages	Never	136 (58.87)
		Occasionally	84 (36.36)
		Once daily	11 (4.76)
		≥2 times daily	0 (0)
Lifestyle Characteristics	Physical exercise*	None	57 (24.78)
		1 per month	64 (27.83)
		>1/month	51 (22.17)
		≥2 times/week	58 (25.22)
	Eating habits*	Unhealthy	27 (11.74)
		Not very healthy	98 (42.61)
		Rather healthy	95 (41.30)
		Very healthy	10 (4.35)
	Hobbies and leisure*	None	42 (18.26)
		Rarely	76 (33.04)
		Occasionally	90 (39.13)
		Regularly	22 (9.57)
	Smart devices use for entertainment*	<2 hours daily	21 (9.13)
		2–4 hours daily	92 (40.00)
		>4–6 hours daily	63 (27.39)
		>6 hours daily	54 (23.48)
	Average hours of sleep*	<4 hours daily	17 (7.39)
		4–6 hours daily	125 (54.35)
		>6–9 hours daily	79 (34.35)
		>9 hours daily	9 (3.91)
	Sleep quality*	Poor	16 (6.96)
		Unsatisfactory	45 (19.57)
		Acceptable	133 (57.83)
		Good	36 (15.65)
	Compliance with religious duties	Poor	16 (6.93)
		Unsatisfactory	37 (16.02)
		Acceptable	84 (36.36)
		Good	94 (40.69)

Note: *The variable has missing values. SD: Standard deviation; IQR: Interquartile range.

**Table 2.** Levels of perceived psychological distress with regards to academic and non-academic stressors.

Subscale	Item	Cronbach's alpha*	Mean (SD) score^†^	High level of perceived psychological distress )score 4–5, n] %[)
Academic life stressors	University environment	0.887	2.59 (1.19)	50 (21.74)
	Science content	0.885	2.85 (1.14)	63 (27.51)
	Efforts to learn	0.879	3.07 (1.28)	96 (41.74)
	Relationship with college mates	0.885	1.72 (1.03)	18 (7.86)
	Relationship with teachers	0.887	1.52 (0.90)	12 (5.24)
	Academic achievement	0.883	3.07 (1.33)	95 (41.48)
Non-academic life stressors	Health status	0.883	1.88 (1.19)	30 (13.04)
	Economic status	0.881	1.78 (1.09)	19 (8.26)
	Home environment	0.881	1.92 (1.23)	34 (14.78)
	Relationship with family	0.880	1.83 (1.20)	32 (13.91)
	Emotional life	0.884	1.94 (1.29)	37 (16.09)
	Relationship with friends	0.878	1.66 (1.08)	19 (8.26)
	Bodily appearance	0.882	2.06 (1.22)	31 (13.48)

Note: *Cronbach's alpha if item is deleted; ^†^scores range from 1–5.

**Table 3.** Academic and non-academic stressors as predictors of psychological co-morbidities.

	Step 1	Step 2	Step 3	Step 4	Step 5
Depression
Academic achievement	4.94	4.05	3.75	3.64	3.34
Relationship with friends		2.98	2.09	1.46	1.33
Emotional life			1.64	1.54	1.52
Health status				1.31	1.17
University environment					1.00
*R^2^*	0.34	0.41	0.43	0.44	0.46
F	116.86*	78.62*	57.52*	45.39*	37.45*
Anxiety
Bodily appearance	3.29	2.43	2.02	1.75	
Relationship with family		2.10	1.91	1.60	
University environment			1.75	1.55	
Health status				1.15	
*R^2^*	0.19	0.26	0.31	0.32	
F	54.66*	39.49*	33.17*	26.77*	
Stress
Academic achievement	4.60	3.86	3.40	2.85	2.59
Health status		2.90	2.15	1.81	1.66
Relationship with family			2.15	2.15	1.80
University environment				1.72	1.57
Bodily appearance					1.19
*R^2^*	0.28	0.36	0.40	0.42	0.43
F	88.02*	63.62*	49.58*	40.98*	33.26*

Note: Stepwise linear regression. Only coefficients with statistically significant correlation are reported. *p < 0.001.

Table 3 demonstrates the magnitude of the impact of each academic and non-academic stressor on psychological co-morbidities. In the first step of the stepwise regression model of depression, ‘academic achievement’ explained 34% of the variance in depression score. The score of explained variance has been increased to 41% after including ‘relationship with friends' into the model. After including emotional life, health status, and university environment, the score of explained variance has been increased to 43%, 44%, and 46%, respectively. In the anxiety stepwise regression model, bodily appearance explained 19% of the variance in anxiety score. The score of explained variance has been increased to 26% after including ‘relationship with family’ in the model. A further increase to 31% and 32% in the score of explained variance have been found after including ‘university environment’ and ‘health status,’ respectively. Concerning the stress, in the first step of stepwise linear regression, the item ‘academic achievement’ showed the highest score of explained variance in stress score (28%). This was followed by ‘health status' that increased the score of explained variance to 36%. Further increases to 40%, 42%, and 43% in the score of explained variance were found after including ‘relationship with family’, ‘university environment’ and ‘bodily appearance,’ respectively.

The multivariate logistic regression analysis revealed that the academic and non-academic stressors score was significantly associated with depression, adjusting for the confounding effect of matching with the first choice in admission (Table 4). Students with higher academic and non-academic stressors score had an increased risk of depression (adjusted Odds Ratio, aOR = 1.13, 95% CI 1.07–1.19). Additionally, students who matched with their first choice in the admission were less likely to have depression.

**Table 4.** Univariate and multivariate logistic regression analysis of different factors on depression among medical students.

Variable	Category	OR	P-value	95% CI		aOR	P-value	95% CI
				LL	UL			LL	UL
Academic and non-academic stressors score		1.15	<0.001*	1.10	1.20	1.13	<0.001*	1.07	1.19
Matching with the first choice in admission	No	Ref				Ref			
	Yes	0.46	0.063	0.20	1.04	0.21	0.023*	0.06	0.81

Note: OR: Odds ratio; aOR: adjusted odds ratio; CI: confidence interval; UL: Upper limit; LL: Lower limit. *Statistically significant.

Multivariate logistic regression analysis indicated that the probability of anxiety risk increased with the academic and non-academic stressors score (aOR = 1.07, 95% CI 1.03–1.12), after adjusting for gender, daily studying hours after school, failure in module(s), eating habit, and working during the study period. Furthermore, the same model showed that having a higher academic and non-academic stressors score, being female, spending more hours for studying after school, failure in module(s), having less healthy eating habits, and working during the study period were associated with a higher chance of anxiety (Table 5).

**Table 5.** Univariate and multivariate logistic regression analysis of different factors on anxiety among medical students.

Variable	Category	OR	P-value	95% CI		aOR	P-value	95% CI
				LL	UL			LL	UL
Academic and non-academic stressors score		1.11	<0.001*	1.07	1.15	1.07	0.002*	1.03	1.12
Gender	Male	Ref				Ref			
	Female	2.47	0.001*	1.45	4.23	3.22	0.003*	1.50	6.90
Daily studying hours after school†		1.66	0.002*	1.20	2.29	1.99	0.003*	1.27	3.14
Failure in module(s)	No	Ref				Ref			
	Yes	3.85	0.047*	1.01	14.62	6.72	0.045*	1.04	43.34
Eating habits‡		0.65	0.018*	0.45	0.93	0.47	0.003*	0.28	0.77
Working during the study period	No	Ref				Ref			
	Yes	0.92	0.874	0.33	2.56	5.02	0.046*	1.03	24.55

Note: OR: Odds ratio; aOR: adjusted odds ratio; CI: confidence interval; UL: Upper limit; LL: Lower limit. *Statistically significant. ^†^Ordinal variable (1: less than 1 hour, 2: 1–2 hours, 3: more than 2–4 hours, and 4: more than 4 hours). ^‡^Ordinal variable (1: unhealthy, 2: not very healthy, 3: rather healthy, and 4: very healthy)

Another multivariate regression analysis concerning the stress was carried out and tabulated (Table 6). The generated model showed that students with a higher academic and non-academic stressors score had more risk of stress (aOR = 1.12, 95% CI 1.08–1.17), after controlling for the confounding effect of smart device use for entrainment.

**Table 6.** Univariate and multivariate logistic regression analysis of different factors on stress among medical students.

Variable	OR	P-value	95% CI		aOR	P-value	95% CI
			LL	UL			LL	UL
Academic and non-academic stressors score	1.13	<0.001*	1.09	1.17	1.12	<0.001*	1.08	1.17
Smart device use for entertainment^†^	1.66	0.001*	1.23	2.23	1.65	0.014*	1.09	2.23

Note: OR: Odds ratio; aOR: adjusted odds ratio; CI: confidence interval; UL: Upper limit; LL: Lower limit. *Statistically significant. †Ordinal variable (1: less than 2 hours daily, 2: 2–4 hours daily, 3: more than 4–6 hours daily, and 4: more than 6 hours daily).

## 4. Discussion

The present study findings revealed a higher prevalence of DAS (55.8%, 45.9%, and 37.7%, respectively) among study participants. These findings are matched with those reported by several studies that reported high prevalence rates of negative emotional states among medical students [22],[41]–[43]. In Saudi Arabia, Kulsoom and Afsar reported that the incidence of depression, anxiety, and stress among medical students were high (43%, 63%, and 41%, respectively) [22]. In Egypt, higher prevalence rates among medical students were reported, 60.2% for depression symptoms, 64.3% for anxiety symptoms and 62.5% for stress symptoms [41]. Iqbal et al. (2015) reported that more than half of medical students in the Institute of Medical Sciences at Bhubaneswar, India had symptoms of depression (51.3%), anxiety (66.9%) and stress (53%) [42]. On the other hand, a Pakistani study reported a much higher prevalence of depression symptoms (71%) and anxiety symptoms (72%) among medical students [43]. In contrast to our results, a previous report indicated that 12.5% of depression prevalent among university students [44]. In some developed countries, a low prevalence of depression, anxiety, and distress symptoms was reported [15]. Western countries pioneered curriculum innovation to alleviate mental stress decades ago, but this concept is comparatively new in Asia, and medical institutions rarely devote sufficient attention to students' wellbeing [45].

There are several academic and non-academic factors potentially associated with such emotional sufferings. Multiple stressors, e.g., learning distress, inadequate lecturer-student relationships, or future concerns, lead to poor psychological wellbeing for most medical students. Other possible stressors contributing to mental health problems among medical students include financial, workload, academic pressure, parent and child relationships, family problems, peer relationships, physical illness, and emotional problems [46],[47]. A study found that depression symptoms were related to unhappiness with life, social loneliness, sleeplessness, and other factors. Similarly, a recent review study identified several factors relate to depression and anxiety symptoms, such as feminine gender, economic status, academic pressure, and others [48]. The present study found that academic achievement was the strongest and most important indicator of depression symptoms, followed by students' relationship with friends, emotional life, and health status. Moreover, bodily appearance was the strongest predictor for students' anxiety, followed by their relationship with family, university environment, and health status. Furthermore, academic achievement was the strongest indicator for stress symptoms, followed by health status, relationship with family, university environment, and bodily appearance. Unlike studies from other countries, economic status was not significantly associated with the psychological morbidities in our findings [2],[49]. This is probably due to the facts that university students in Saudi Arabia do not pay for their tuition; in fact, they receive a monthly allowance.

Medical study is indeed a heavily loaded discipline and unbearable to some students and poor academic performance was linked to psychological distress, including symptoms of anxiety and depression [50]. Therefore, suffering students might view academic achievement as a source of their perceived distress. Saudi and Pakistani studies reported that students believed that the massive course content, frequent assessments, long duty hours, future apprehension and fewer recreational activities contributed to heightened torment [21],[43]. A Malaysian study documented academic burden, failure to enjoy daily activities, loss of focus, and students relationship were the few major linked with depression symptoms among first-year medical students [51]. Another Malaysian study stated that medical students' depression and anxiety symptoms were related to low academic performance [52]. A previous study carried out in Saudi Arabia showed that depression symptoms were linked to poor academic performance [10].

Literature has described that bodily appearance and its relationship with anxiety symptoms among medical students. A study found that 78.8% of medical students were dissatisfied with some aspect of their appearance and another study reported 32.5% of medical students were concerned about their bodily appearance unrelated to the weight [53],[54]. In addition, up to 5.8% of the medical students met the DSM-IV criteria of body dysmorphic disorder [53],[54]. Students dissatisfied with their appearance tended to have a high social anxiety score [53]. This was consistent with our finding that showed bodily appearance was an important player in the occurrence of anxiety.

It is a widely known truth that the academic workload in medical education is significantly greater. DAS symptoms impede medical students' academic careers and, later, their social lives. While some stress is necessary for optimum performance, prolonged stress can impair an individual's capacity to deal [55]. So appropriate controlling measures are required to minimize these career hampering factors. Medical education is associated with DAS symptoms, and it is a deep-rooted problem that occurs at some point in every medical student's life; therefore, it should be identified and treated at an early stage. Several suggestions have been recommended in the literature for coping with the DAS symptoms for medical students. Courses on stress management and wellness should be included in the curriculum so that students can relax and focus on their studies. Study skills and time management should be highlighted and handled regularly. A student support section should counsel and inform students how to cope with the demanding medical environment and become competent professionals [56]. In addition, students should be motivated to use stress-reduction tactics and participate in healthy recreational and leisure activities [56]. Faculty members should provide regular feedback to their students and collaborate to identify effective techniques for improving student performance. Students should participate in social activities that will help them build a sense of teamwork [13]. Students should be encouraged to have a healthy lifestyle (healthy food, frequent exercise, aerobics, appropriate sleep, and meditation) to improve their mental and physical health [57]. Most importantly, instead of ignoring their DAS symptoms, students should be encouraged to talk about them. Students should also be taught various effective and practical stress management techniques [13]. Students should be encouraged to regularly communicate with their families and friends to build a solid support network [13]. Rehman et al. (2017) suggested that advantageous for medical students to be aware of and employ spiritual wellbeing as a coping mechanism for stress and depression symptoms [58].

In the present study, the authors did not include the socio-demographic, academic, and lifestyle factors in the stepwise regression analysis that solely evaluated the panel of reported stressors against DAS. For instance, the authors did not account for the impact of gender on bodily appearance. In addition, some non-academic stressors including bodily appearance and academic achievement were not clearly defined. Furthermore, the authors did not estimate the sample size beforehand. Our study, in addition, was limited by convenience sampling. Therefore, the study lacks the generalizability of the generated data to the population at large. A questionnaire-based study is suitable to estimate the prevalence of psychological morbidities; nevertheless, it is inappropriate to ascertain causal pathways between variables. Besides, the tool used in this study provides an adequate screening for the psychological morbidities; however, further clinical evaluation is required to establish the diagnosis.

## 5. Conclusions

In conclusion, our findings explained the high rate of DAS among medical students. Besides, academic achievement was the noteworthy factor that was linked to symptoms of depression and stress, while body image was associated anxiety symptoms amongst medical students.

## Recommendation

It is advised that key negative emotional stressors be identified in order to control such stressors in a timely manner. There is a need to bring changes in the educational environment of the medical institutes to reduce their academic burden. Medical education is such a profession that needs extensive studies and deep concentration for getting success. It is recommended that medical students be aware of all risk factors and have access to psychological counseling during their early academic years. Students' counseling unit should assist them in mitigating the obstacles that may threaten their bright career prospects.

## References

[1] Stanley N, Manthorpe J (2001). Responding to students' mental health needs: Impermeable systems and diverse users. J Ment Health.

[2] Dyrbye LN, Thomas MR, Shanafelt TD (2006). Systematic review of depression, anxiety, and other indicators of psychological distress among U.S. and Canadian medical students. Acad Med.

[3] Guthrie EA, Black D, Shaw CM (1997). Psychological Stress in Medical Students: A Comparison of Two Very Different University Courses. Stress Med.

[4] Sherina MS, Rampal L, Kaneson N (2004). Psychological stress among undergraduate medical students. Med J Malaysia.

[5] GBD 2015 Disease and Injury Incidence and Prevalence Collaborators (2016). Global, regional, and national incidence, prevalence, and years lived with disability for 310 diseases and injuries, 1990–2015: a systematic analysis for the Global Burden of Disease Study 2015. Lancet.

[6] Ibrahim AK, Kelly SJ, Adams CE (2013). A systematic review of studies of depression prevalence in university students. J Psychiatr Res.

[7] Ludwig AB, Burton W, Weingarten J (2015). Depression and stress amongst undergraduate medical students. BMC Med Educ.

[8] Alharbi H, Almalki A, Alabdan F (2018). Depression among medical students in Saudi medical colleges: a cross-sectional study. Adv Med Educ Pract.

[9] Albajjar MA, Bakarman MA (2019). Prevalence and correlates of depression among male medical students and interns in Albaha University, Saudi Arabia. J Family Med Prim Care.

[10] Aboalshamat K, Hou XY, Strodl E (2015). Psychological well-being status among medical and dental students in Makkah, Saudi Arabia: a cross-sectional study. Med Teach.

[11] Hamasha AA, Kareem YM, Alghamdi MS (2019). Risk indicators of depression among medical, dental, nursing, pharmacology, and other medical science students in Saudi Arabia. Int Rev Psychiatr.

[12] AlShamlan NA, AlShamlan RA, AlShamlan AA (2020). Prevalence of depression and its associated factors among clinical-year medical students in Eastern Province, Saudi Arabia. Postgrad Med J.

[13] Khanagar SB, Al-Ehaideb A, Jamleh A (2021). Psychological Distress among Undergraduate Dental Students in Saudi Arabia and Its Coping Strategies-A Systematic Review. Healthcare (Basel).

[14] AlJaber MI (2020). The prevalence and associated factors of depression among medical students of Saudi Arabia: A systematic review. J Family Med Prim Care.

[15] Hope V, Henderson M (2014). Medical student depression, anxiety and distress outside North America: a systematic review. Med Educ.

[16] Ibrahim N, Al-Kharboush D, El-Khatib L (2013). Prevalence and Predictors of Anxiety and Depression among Female Medical Students in King Abdulaziz University, Jeddah, Saudi Arabia. Iran J Public Health.

[17] Inam SB (2007). Anxiety and Depression among Students of a Medical College in Saudi Arabia. Int J Health Sci (Qassim).

[18] Alahmadi AM (2019). Prevalence of Anxiety Among College and School Students in Saudi Arabia: A systematic review. J Health Inf Dev Countries.

[19] Nuqali A, Al Nazzawi H, Felmban S (2018). Assessing the Correlation between Medical Students' Psychological Distress and Their Academic Performance in Makkah, Saudi Arabia. Creative Educ.

[20] Shadid A, Shadid AM, Shadid A (2020). Stress, Burnout, and Associated Risk Factors in Medical Students. Cureus.

[21] Gazzaz ZJ, Baig M, Al Alhendi BSM (2018). Perceived stress, reasons for and sources of stress among medical students at Rabigh Medical College, King Abdulaziz University, Jeddah, Saudi Arabia. BMC Med Educ.

[22] Kulsoom B, Afsar NA (2015). Stress, anxiety, and depression among medical students in a multiethnic setting. Neuropsychiatr Dis Treat.

[23] Bramness JG, Fixdal TC, Vaglum P (1991). Effect of medical school stress on the mental health of medical students in early and late clinical curriculum. Acta Psychiatr Scand.

[24] Stewart SM, Betson C, Lam TH (1997). Predicting stress in first year medical students: a longitudinal study. Med Educ.

[25] Tyssen R, Vaglum P, Grønvold NT (2001). Factors in medical school that predict postgraduate mental health problems in need of treatment. A nationwide and longitudinal study. Med Educ.

[26] El-Gilany AH, Amr M, Hammad S (2008). Perceived stress among male medical students in Egypt and Saudi Arabia: effect of sociodemographic factors. Ann Saudi Med.

[27] Abdulghani HM, AlKanhal AA, Mahmoud ES (2011). Stress and its effects on medical students: a cross-sectional study at a college of medicine in Saudi Arabia. J Health Popul Nutr.

[28] Alzahrani AM, Hakami A, AlHadi A (2020). The interplay between mindfulness, depression, stress and academic performance in medical students: A Saudi perspective. PLoS One.

[29] Bahhawi TA, Albasheer OB, Makeen AM (2018). Depression, anxiety, and stress and their association with khat use: a cross-sectional study among Jazan University students, Saudi Arabia. Neuropsychiatr Dis Treat.

[30] Aboalshamat K, Jawhari A, Alotibi S (2017). Relationship of self-esteem with depression, anxiety, and stress among dental and medical students in Jeddah, Saudi Arabia. J Int Med Dent.

[31] Beiter R, Nash R, McCrady M (2015). The prevalence and correlates of depression, anxiety, and stress in a sample of college students. J Affect Disord.

[32] Shaikh BT, Kahloon A, Kazmi M (2004). Students, stress and coping strategies: a case of Pakistani medical school. Educ Health (Abingdon).

[33] Dyrbye LN, Thomas MR, Huntington JL (2006). Personal life events and medical student burnout: a multicenter study. Acad Med.

[34] Dyrbye LN, Thomas MR, Harper W (2009). The learning environment and medical student burnout: a multicentre study. Med Educ.

[35] Santen SA, Holt DB, Kemp JD (2010). Burnout in medical students: examining the prevalence and associated factors. South Med J.

[36] Crawford JR, Henry JD (2003). The Depression Anxiety Stress Scales (DASS): normative data and latent structure in a large non-clinical sample. Br J Clin Psychol.

[37] Lovibond SH, Lovibond PF (1995). Manual for the depression anxiety stress scales (2nd ed.).

[38] Sinclair SJ, Siefert CJ, Slavin-Mulford JM (2012). Psychometric evaluation and normative data for the depression, anxiety, and stress scales-21 (DASS-21) in a nonclinical sample of U.S. adults. Eval Health Prof.

[39] Daza P, Novy DM, Stanley MA (2002). The Depression Anxiety Stress Scale-21: Spanish Translation and Validation with a Hispanic Sample. J Psychopathol Behav Assess.

[40] Byrne DG, Davenport SC, Mazanov J (2007). Profiles of adolescent stress: the development of the adolescent stress questionnaire (ASQ). J Adolesc.

[41] Abdel Wahed WY, Hassan SK (2017). Prevalence and associated factors of stress, anxiety and depression among medical Fayoum University students. Alex J Med.

[42] Iqbal S, Gupta S, Venkatarao E (2015). Stress, anxiety and depression among medical undergraduate students and their socio-demographic correlates. Indian J Med Res.

[43] Azim SR, Baig M (2019). Frequency and perceived causes of depression, anxiety and stress among medical students of a private medical institute in Karachi: a mixed method study. J Pak Med Assoc.

[44] Rehman R, Fatima K, Hussain M (2021). Association between depression and health risk behaviors among university students, Karachi, Pakistan. Cogent Psychology.

[45] Oku A, Owoaje E, Oku O (2015). Prevalence of stress, stressors and coping strategies among medical students in a Nigerian medical school. Afr J Med Health Sci.

[46] Niemi PM, Vainiomaki PT (2006). Medical students' distress--quality, continuity and gender differences during a six-year medical programme. Med Teach.

[47] Sharifirad G, Marjani A, Abdolrahman C (2012). Stress among Isfahan medical sciences students. J Res Med Sci.

[48] Mirza AA, Baig M, Beyari GM (2021). Depression and Anxiety Among Medical Students: A Brief Overview. Adv Med Educ Pract.

[49] Yusoff MSB, Yee LY, Wei LH (2011). A study on stress, stressors and coping strategies among Malaysian medical students. Int J Stud Res.

[50] Yeh YC, Yen CF, Lai CS (2007). Correlations between Academic Achievement and Anxiety and Depression in Medical Students Experiencing Integrated Curriculum Reform. Kaohsiung J Med Sci.

[51] Yusoff MSB, Rahim AFA, Yaacob MJ (2011). The Prevalence of Final Year Medical Students with Depressive Symptoms and Its Contributing Factors. Int Med J.

[52] Saravanan C, Wilks R (2014). Medical students' experience of and reaction to stress: the role of depression and anxiety. Scientific World J.

[53] Liao Y, Knoesen NP, Deng Y (2010). Body dysmorphic disorder, social anxiety and depressive symptoms in Chinese medical students. Soc Psychiatry Psychiatr Epidemiol.

[54] Taqui AM, Shaikh M, Gowani SA (2008). Body Dysmorphic Disorder: gender differences and prevalence in a Pakistani medical student population. BMC Psychiatr.

[55] Behere SP, Yadav R, Behere PB (2011). A comparative study of stress among students of medicine, engineering, and nursing. Indian J Psychol Med.

[56] Tahir M, Butt MW, Gul S (2020). Factors contributing to stress and anxiety in undergraduate medical students. Professional Med J.

[57] Dunn LB, Iglewicz A, Moutier C (2008). A conceptual model of medical student well-being: promoting resilience and preventing burnout. Acad Psychiatr.

[58] Rehman R, Katpar S, Hussain M (2017). A comparison between wellness awareness among medical students. J Pak Med Assoc.

